# Supplementary material for: Stromal-driven and Amyloid β-dependent induction of neutrophil extracellular traps modulates tumor growth
Source: Nat Commun. 2021 Jan 29;12:683. doi: 10.1038/s41467-021-20982-2 (PMC7846803; doi:10.1038/s41467-021-20982-2)
Supplement: Supplementary file 1 — Supplementary Information [file 41467_2021_20982_MOESM1_ESM.pdf]

SUPPLEMENTARY INFORMATION FILE

Stromal-driven and Amyloid β-dependent induction of neutrophil extracellular traps modulates tumour growth

Hafsa Munir[1], James O. Jones[1,2], Tobias Janowitz[3,4,5], Markus Hoffmann[6], Maximilien Euler[6], Carla P. Martins[1], Sarah J. Welsh[2], Jacqueline D. Shields[1*].

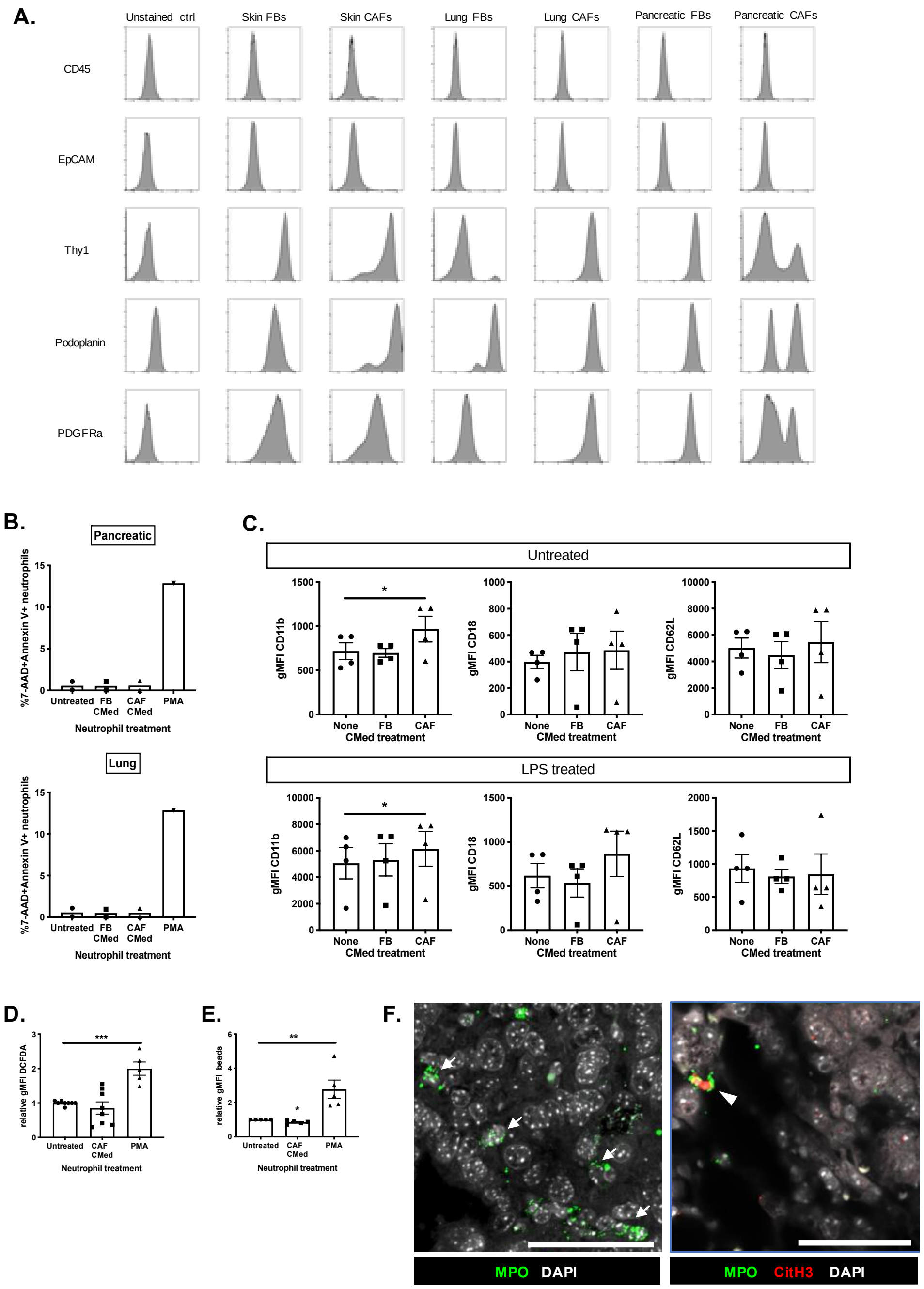

Sup Fig. 1. Neutrophil activation and function are unaltered by short term stimulation with CAF-derived factors. (**A**) Characterization of skin, lung and pancreatic FBs and CAFs by flow cytometry showing that the cells express the classic CAF markers (Thy1, Podoplanin, PDGFRα) on the surface and lack expression of tumor cell (EpCAM) and immune cell (CD45) markers. (**B**) Quantification of neutrophil death after treatment with FB CMed, CAF CMed from lung or pancreas or PMA based on expression of Annexin V and 7-AAD by flow cytometry. (**C**) Quantification of CD11b, CD18 and CD62L expression on neutrophils after treatment with lung FB or CAF CMed with and without simulation with LPS by flow cytometry. (**D**) Quantification of ROS production by neutrophils after treatment with lung CAF CMed or PMA based on the levels of DCFDA by flow cytometry. (**E**) The phagocytic capacity of neutrophils after lung CAF CMed or PMA treatment based on uptake of fluorescent 1µm beads assessed by flow cytometry. (**F**) Representative confocal image of neutrophils (arrows) and NETs (arrowheads) in murine lung cancer, scale bar 50µm. Data are mean ± SEM; * = $p<0.05$, ** = $p<0.01$ and *** = $p<0.001$ using (C) paired t-test (comparing each condition) and (D-E) one-

way ANOVA with a Dunnett post hoc test. Assays were performed on (A) n=1, (B) n=2, (C) n=4, (D) n=8 (Untreated and CAF CMed) and n=5 (PMA), (E) n=5 and (F) Representative of n=3 independent experiments.

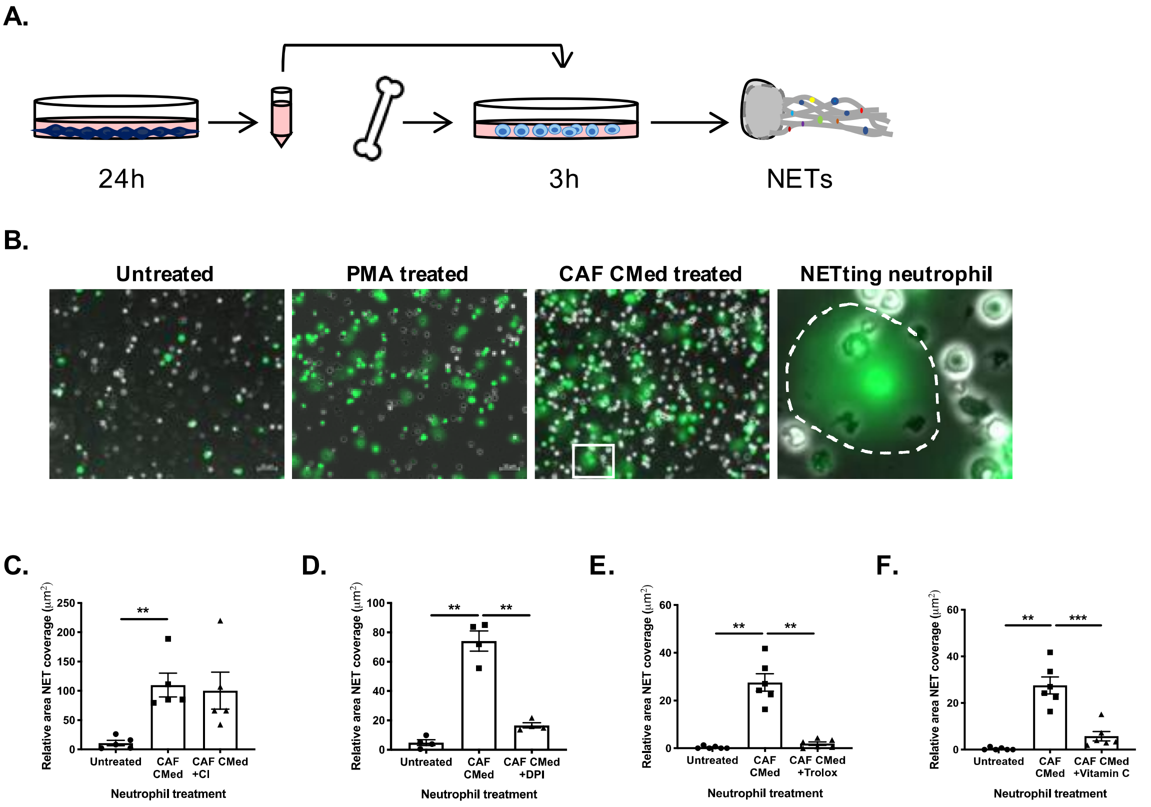

Sup Fig. 2. CAF-induced NETosis is a ROS-dependent, autophagy independent response. (**A**) Schematic of NETosis assay (**B**) Representative phase contrast images of SYTOX green stained dead neutrophils and NETs after 3h treatment with PMA or pancreatic CAF CMed. Inset; zoomed image of a NET (green haze) outlined with a dashed white line. Quantification of the relative NET coverage of neutrophils treated with lung CAF CMed with or without treatment with (**C**) Chloroquine (Cl), (**D**) Diphenyleneiodonium (DPI), (**E**) Trolox and (**F**) Vitamin C. Data are mean ± SEM; * = $p<0.05$, ** = $p<0.01$ and *** = $p<0.001$ using (C-F) one-way ANOVA with a Tukey post hoc test. Assays were performed on (B) Representative of n=7, (C) n=5, (D) n=4, (E-F) n=2 (in triplicate) independent experiments. Scale bars correspond to 50µm.

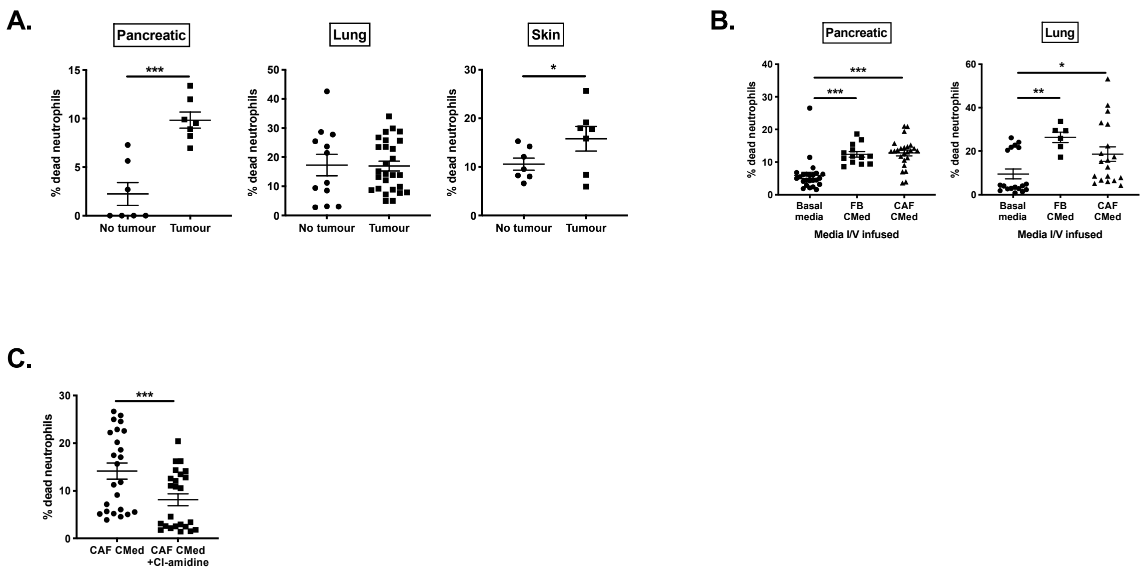

Sup Fig. 3. CAF-derived factors drive NETosis systemically. (**A**) Quantification of the percentage death of bone marrow neutrophils taken from mice with pancreatic, lung or skin tumors. (**B**) Quantification of percentage death of bone marrow neutrophils taken from wild type mice intravenously infused with pancreatic or lung FB or CAF CMed 24h before performing the NETosis assay. (**C**) Quantification of percentage death and relative number of bone marrow neutrophils taken from mice intravenously infused with lung CAF CMed with or without pre-treatment with Cl-amidine for 24h. Data are mean ± SEM; * = $p<0.05$, ** = $p<0.01$ and *** = $p<0.001$ using (A and C) paired t-test and (B) one-way ANOVA with Dunnett post hoc test. Assays were performed on (A) n=4 (in duplicate), n=7 (in quadruplet) and n=7 for pancreatic, lung and skin tumor bearing mice respectively, (B) n=8, 4 and 8 (Pancreatic for basal media, FB CMed and CAF CMed treatment, respectively. All performed in triplicate) and n=9, 6 and 9 (Lung for basal media, FB CMed and CAF CMed treated, respectively. Performed in duplicate for basal media and CAF CMed and only a single time for FB CMed treated), (C) n=8 (in triplicate) independent experiments.

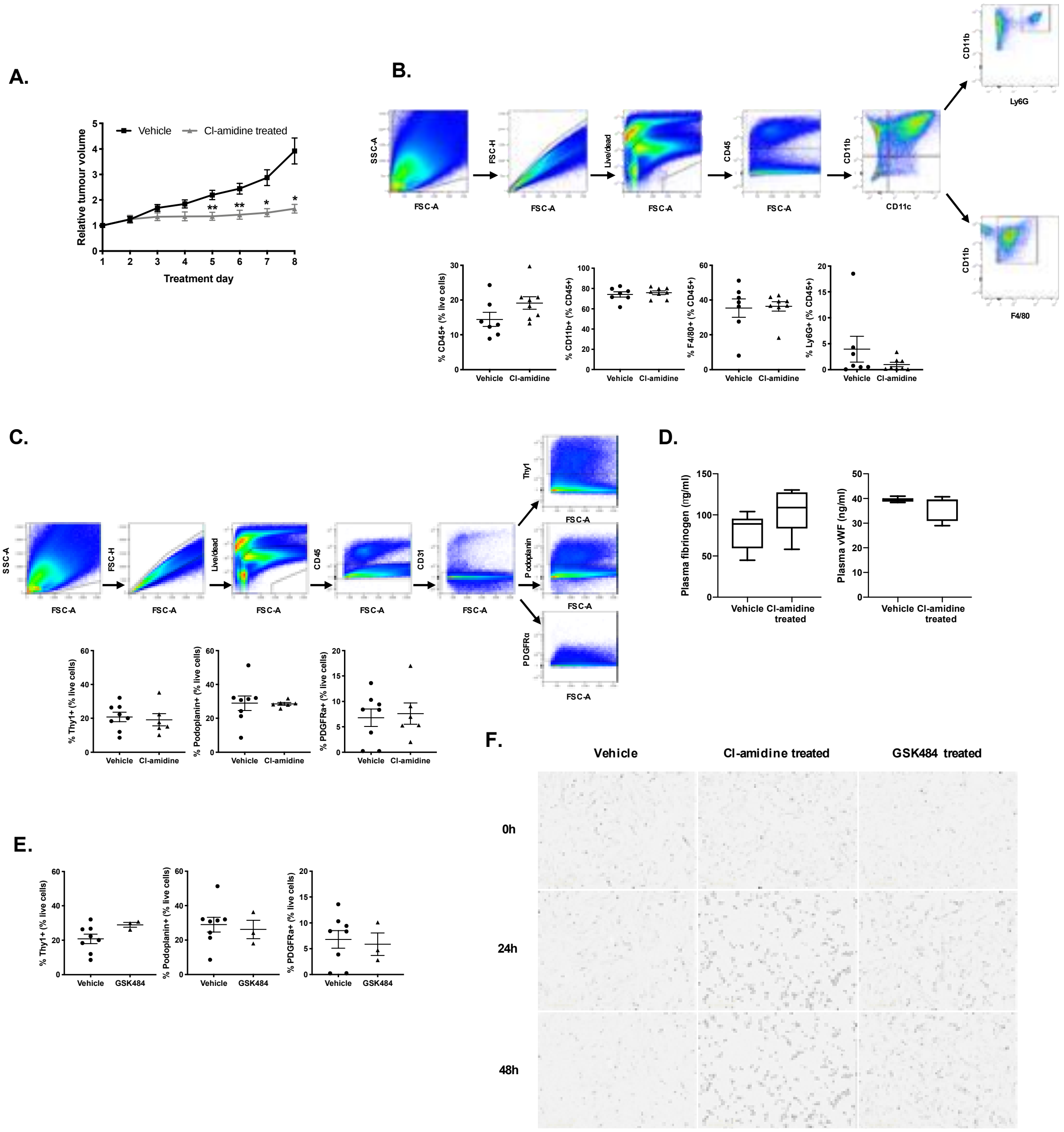

Sup Fig. 4. **Treatment of skin tumor bearing mice with a broad-spectrum PAD inhibitor reduces tumor growth.** (**A**) Relative volume of skin tumors on mice treated with vehicle or Cl-amidine over 8d. (**B**) Flow cytometric analysis of the percentage immune cells (CD45$^+$), myeloid cells (CD11b$^+$), macrophages (F4/80$^+$) and neutrophils (Ly6G$^+$) in the tumors treated with Cl-amidine. (**C**) Flow cytometric analysis of CAF populations in the tumors treated with Cl-amidine based on the percentage Thy1, Podoplanin and PDGFRα positive cells. (**D**) The levels of clotting factors (fibrinogen and von Willerbrand factor; vWF) in the plasma of skin tumor bearing mice treated with vehicle or Cl-amidine. (**E**) Flow cytometric analysis of CAF populations in the tumors treated with GSK484 based on the percentage Thy1, Podoplanin and PDGFRα positive cells. (**F**) Representative phase contrast images of skin tumor cells after treatment with vehicle, 100µM Cl-amidine or 10µM GSK484 over 48h. Bar graphs are mean ± SEM; * = $p<0.05$ and ** = $p<0.01$ using (A) a Mann-Whitney test. Box and whiskers graph- line: median, box: upper and lower quartiles, whiskers: maxima and minima. Assays were performed on (A and B) n=7 (Vehicle) and n=8 (Cl-amidine), (C) n=8 (Vehicle) and n=6 (Cl-amidine) (D) n=6 (both conditions) for Fibrinogen and n=7 (both conditions) for vWF, (E) n=8 (Vehicle) and n=4 (GSK484) in duplicate for all markers, (F) n=3 (Vehicle) and n=2 (Cl-amidine and GSK484) (in duplicate) independent experiments.

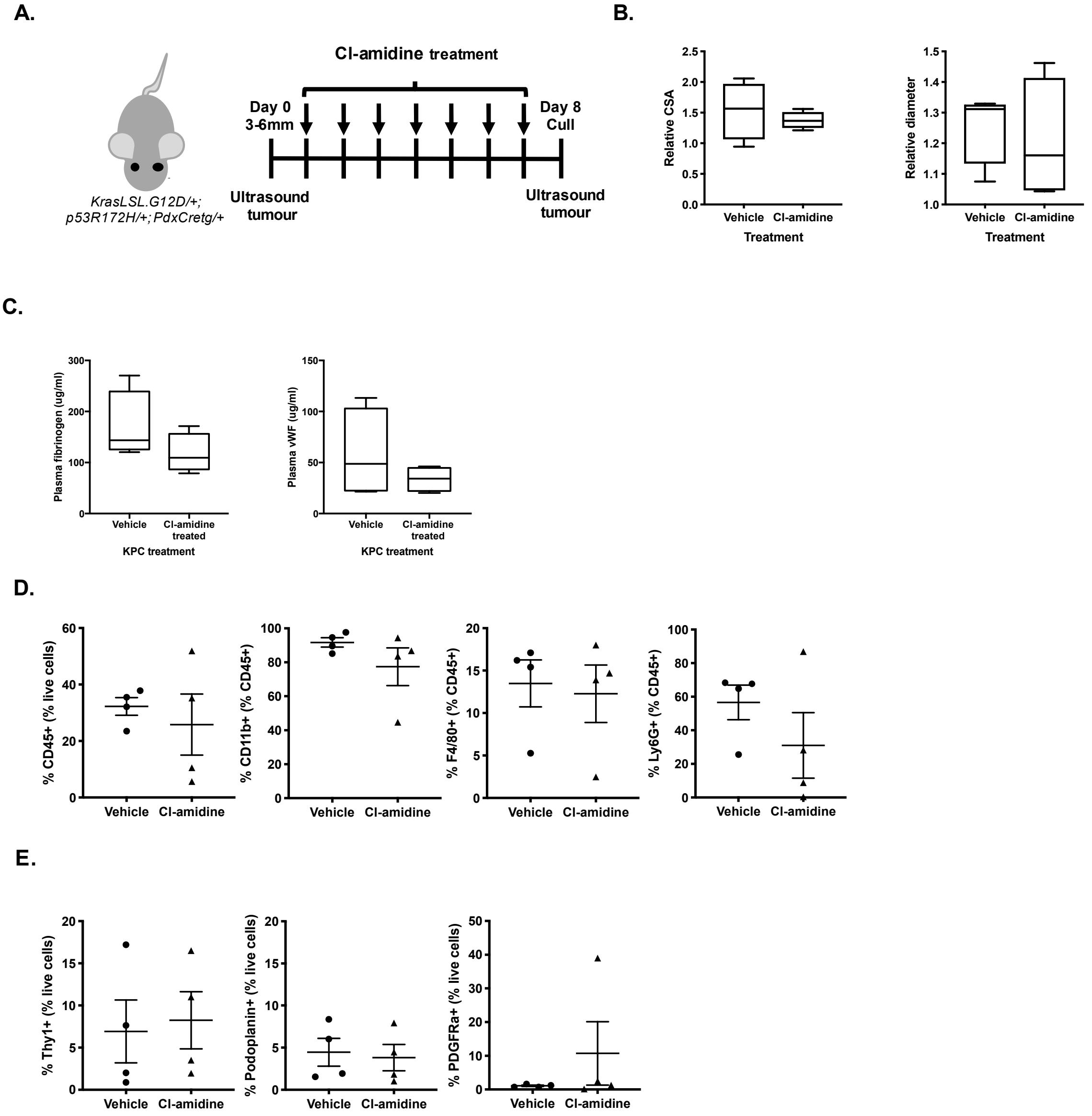

Sup Fig. 5. Treatment of pancreatic tumor bearing mice with a broad-spectrum PAD inhibitor has no effect on tumor growth. (**A**) Schematic of Cl-amidine treatment regime for pancreatic tumor bearing mice. (**B**) Relative cross-sectional area (CSA) and diameter of tumors treated with vehicle or Cl-amidine over 8d. (**C**) The levels of clotting factors (fibrinogen and von Willerbrand factor; vWF) in the plasma of pancreatic tumor bearing mice treated with vehicle or Cl-amidine. (**D**) Flow cytometric analysis of the percentage immune cells (CD45$^+$), myeloid cells (CD11b$^+$), macrophages (F4/80$^+$) and neutrophils (Ly6G$^+$) in the tumor. (**E**) Flow cytometric analysis of CAF populations in the tumor based on the percentage Thy1, Podoplanin and PDGFRα positive cells. Assays were performed on n=4 independent experiments. Bar graphs are mean ± SEM. Box and whiskers graph-line: median, box: upper and lower quartiles, whiskers: maxima and minima.

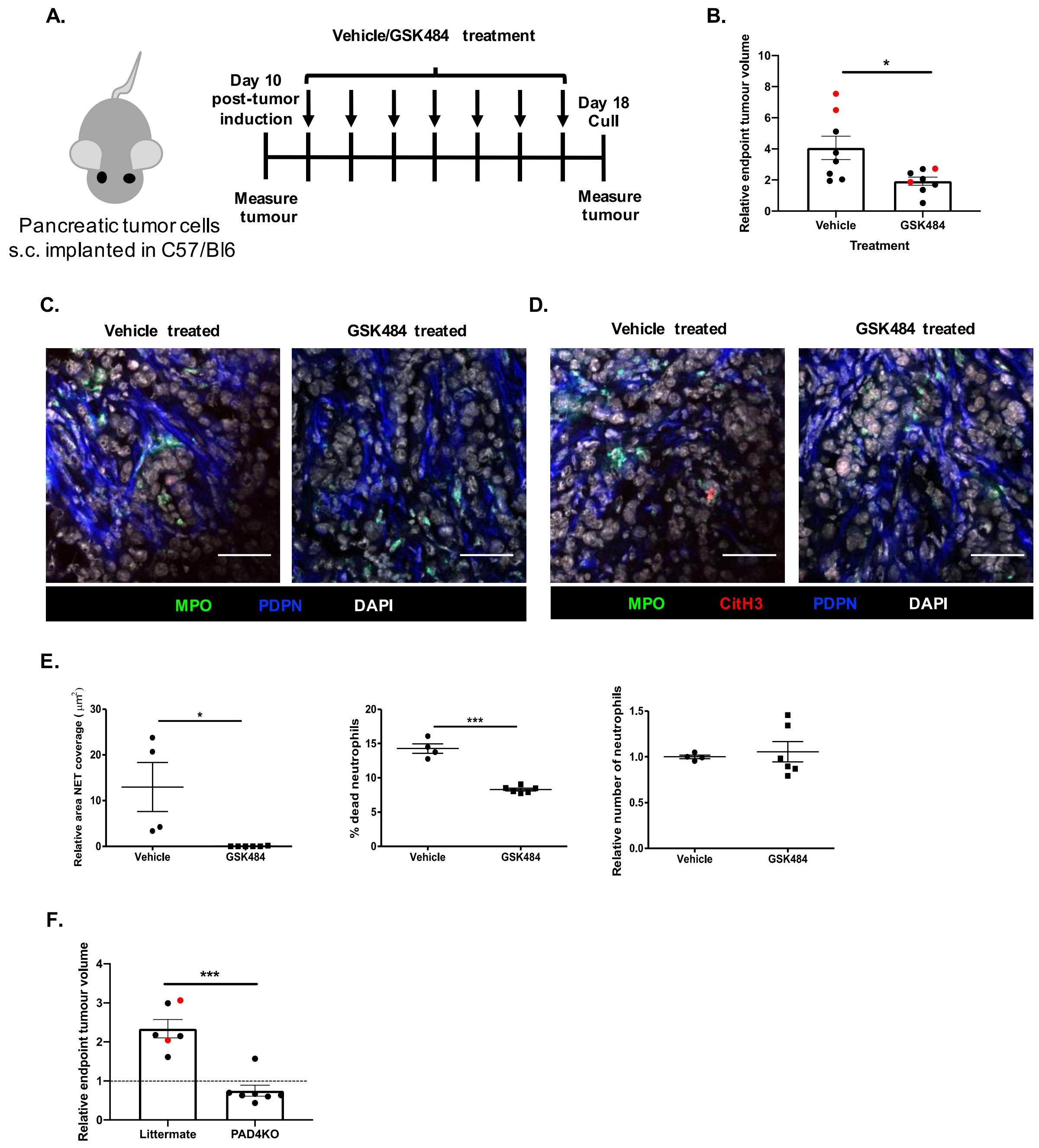

Sup Fig. 6. Inhibiting t-NETosis suppresses growth of subcutaneous (s.c.) pancreatic tumors *in vivo*. (**A**) Schematic of GSK484 treatment regime for s.c. pancreatic tumor-bearing mice. (**B**) Relative endpoint volume of s.c. pancreatic tumors in mice treated with vehicle or GSK484 after 8d (volume compared to the pre-treatment tumor volume – indicated by dotted line). Red dots represent mice reaching legal endpoint prior to the end of the 8d treatment (within 5-7d of treatment). (**C**) Representative confocal images of neutrophils and CAFs in vehicle or GSK484 treated s.c. pancreatic tumors (MPO, green; PDPN, blue; DAPI, white). (**D**) Representative confocal images of NETting neutrophils and CAFs in vehicle or GSK484 treated s.c. pancreatic tumors (MPO, green; CitH3, red; PDPN, blue; DAPI, white). Scale bars are 50µm. (**E**) Quantification of relative NET coverage, percentage death and relative number of bone marrow neutrophils taken from mice with s.c. pancreatic tumors. (**F**) Relative endpoint volume of s.c. pancreatic tumors in PAD4-/- or WT littermate mice after 8d (compared to the tumor volume at day 10 post-induction indicated by dotted line). Red dots indicate mice reaching legal endpoint prior the end of the 8d (within 5-7d). Data are mean ± SEM; * = p<0.05,

** = $p<0.01$ and *** = $p<0.001$ using (B, E-F) unpaired t-test. Assays were performed on (B) n=8 mice, (C-D) Representative of n=3, (E) n=2 or 3 vehicle and GSK484 treated mice respectively (in duplicate) and (F) n=6-7 mice. Scale bars are 50µm.

Figure 7

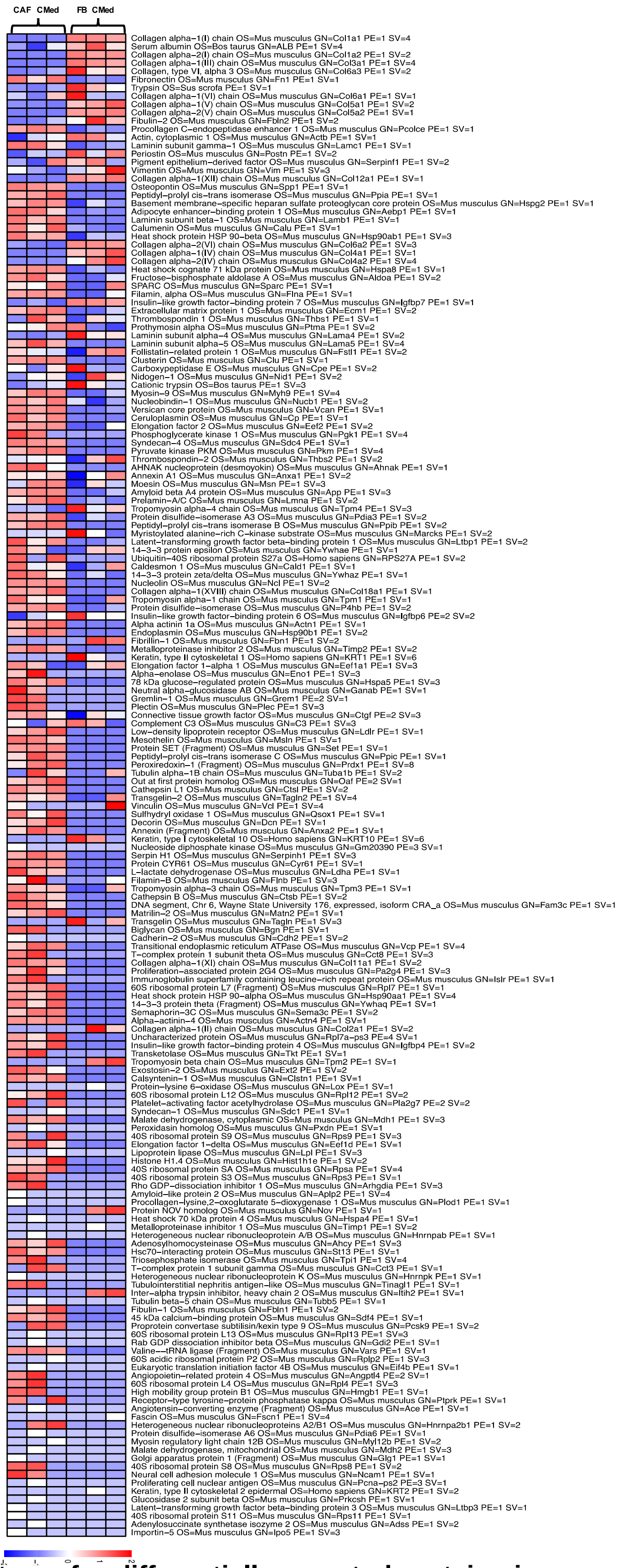

Sup Fig. 7. Enlarged heatmap for differentially secreted proteins in pancreatic FB and CAF CMed analyzed by mass spectrometry. The relative intensity of each protein is indicated by the coloured bar under the heatmap. The assay was performed on n=3 samples.

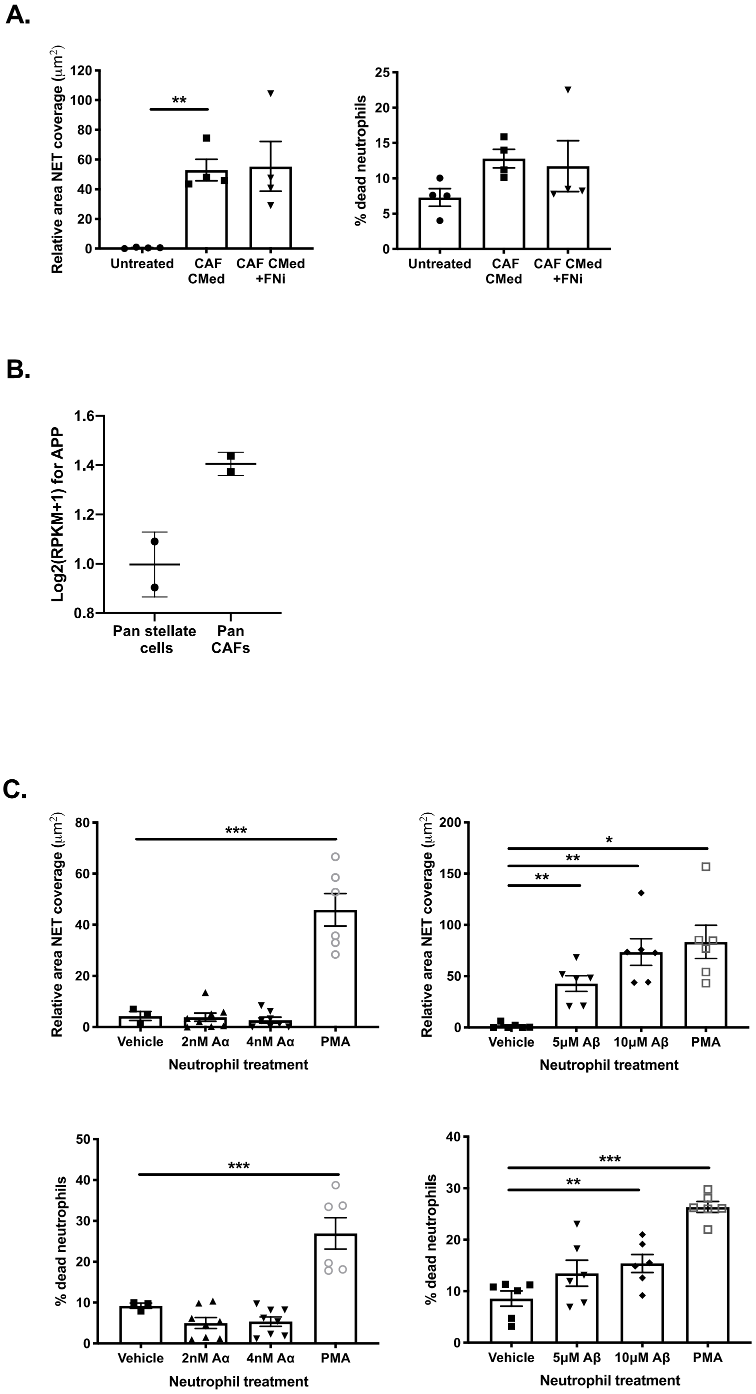

Sup Fig. 8. Amyloid β is sufficient to drive NETosis *in vitro*. (**A**) Quantification of the relative NET coverage and percentage death of neutrophils treated with pancreatic CAF CMed with or without a fibronectin inhibitor (FNi). (**B**) Log2(reads per kilobase transcript per million map reads; RPKM+1) expression of the app gene in pancreatic (pan) CAFs normalized to pancreatic (pan) stellate cells from publicly available data *(2)*. (**C**) Quantification of the relative NET coverage and percentage death of neutrophils treated with recombinant Amyloid α (Aα), recombinant Amyloid β (Aβ) or PMA for 3h. Data are mean ± SEM; * = p<0.05, ** = p<0.01 and *** = p<0.001 using (A) paired t-test and (C) one-way ANOVA with a Dunnett post hoc test. Assays were performed on (A) n=2 (in duplicate), (C) n=3 (in duplicate or triplicate) independent experiments.

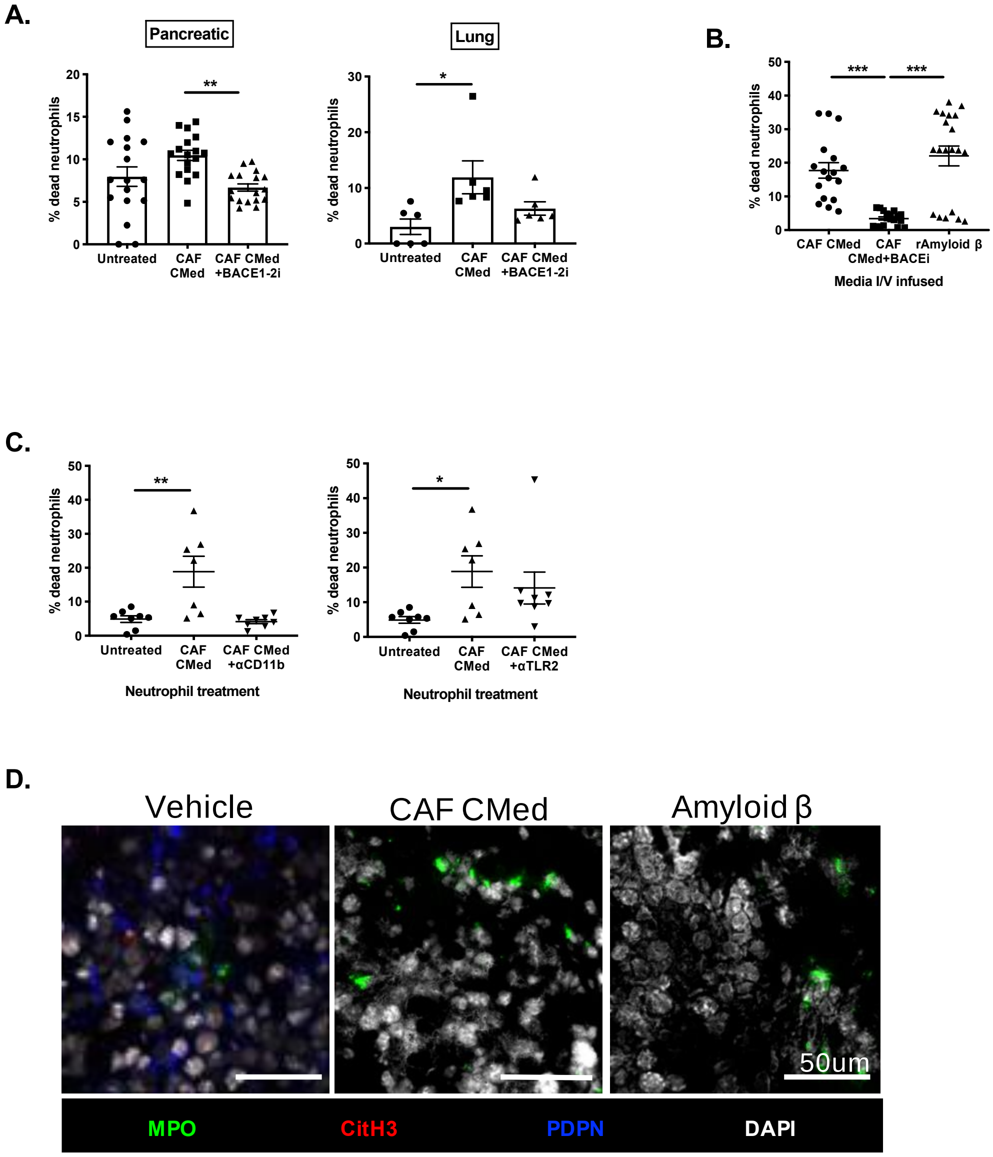

Sup Fig. 9. Inhibition of Amyloid β secretion by CAFs suppresses NETosis. (**A**) Quantification of percentage death of neutrophils treated with pancreatic and lung CAF CMed generated with or without 24h pre-treatment of the CAFs with β-secretases 1 and 2 (BACE1-2) inhibitor.(**B**) Quantification of the percentage death of bone marrow neutrophils taken from wild type mice 24h after intravenous infusion with pancreatic CAF CMed taken from cells pre-treated with or without BACE1-2 inhibitor for 24h or recombinant Amyloid β. (**C**) Quantification of cell death after 3h treatment with pancreatic CAF CMed with or without CD11b or TLR2 blocking antibodies. (**D**) Representative confocal images showing neutrophils (myeloperoxidase (MPO; in green), CAFs (podoplanin, in blue) and counterstained with DAPI (in white) of orthotopically implanted B16.F10 tumor cells with vehicle, CAF CMed or recombinant Amyloid β treatment. Data are mean ± SEM; * = $p<0.05$, ** = $p<0.01$ and *** = $p<0.001$ using (A-B) one-way ANOVA with a Tukey post hoc test and (C) one-way ANOVA with a Dunnett post hoc test. Assays were performed on (A) n=6 (in triplicate), (B) n=7 (in duplicate or triplicate), (C) n=3 (in triplicate) and (D) Representative of n=3 independent experiments. Scale bars are 50µm.

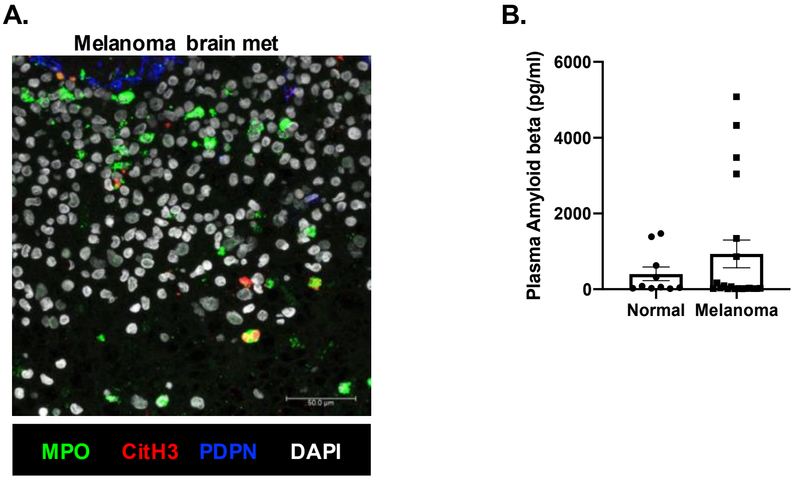

Sup Fig. 10. Amyloid β driven t-NETosis is conserved in the human disease. (**A**) Representative confocal images of NETting neutrophils and CAFs in a human brain metastasis (MPO, green; CitH3, red; podoplanin, blue; DAPI, white). (**B**) Quantification of Amyloid β in plasma from melanoma patients and healthy controls. Data are mean ± SEM. Assays were performed on (A) n=1 brain metastasis and (B) n=10 (Normal) and n=19 (Melanoma) independent experiments. Scale bars are 50µm.
