# Supplementary material for: Stromal-driven and Amyloid β-dependent induction of neutrophil extracellular traps modulates tumor growth
Source: Nat Commun. 2021 Jan 29;12:683. doi: 10.1038/s41467-021-20982-2 (PMC7846803; doi:10.1038/s41467-021-20982-2)
Supplement: Supplementary file 3 — Reporting Summary [file 41467_2021_20982_MOESM3_ESM.pdf]

nature research

Corresponding author(s): Jacqueline D. Shields

Last updated by author(s): Dec 2, 2020

Reporting Summary

Nature Research wishes to improve the reproducibility of the work that we publish. This form provides structure for consistency and transparency in reporting. For further information on Nature Research policies, see our Editorial Policies and the Editorial Policy Checklist.

Statistics

For all statistical analyses, confirm that the following items are present in the figure legend, table legend, main text, or Methods section.

n/a	Confirmed	
☐	☒	The exact sample size (n) for each experimental group/condition, given as a discrete number and unit of measurement
☐	☒	A statement on whether measurements were taken from distinct samples or whether the same sample was measured repeatedly
☐	☒	The statistical test(s) used AND whether they are one- or two-sided<br>*Only common tests should be described solely by name; describe more complex techniques in the Methods section.*
☒	☐	A description of all covariates tested
☐	☒	A description of any assumptions or corrections, such as tests of normality and adjustment for multiple comparisons
☐	☒	A full description of the statistical parameters including central tendency (e.g. means) or other basic estimates (e.g. regression coefficient) AND variation (e.g. standard deviation) or associated estimates of uncertainty (e.g. confidence intervals)
☐	☒	For null hypothesis testing, the test statistic (e.g. F, t, r) with confidence intervals, effect sizes, degrees of freedom and P value noted<br>*Give P values as exact values whenever suitable.*
☒	☐	For Bayesian analysis, information on the choice of priors and Markov chain Monte Carlo settings
☒	☐	For hierarchical and complex designs, identification of the appropriate level for tests and full reporting of outcomes
☒	☐	Estimates of effect sizes (e.g. Cohen's d, Pearson's r), indicating how they were calculated

Our web collection on statistics for biologists contains articles on many of the points above.

Software and code

Policy information about availability of computer code

Data collection	Zen Blue and Zen Black for image acquisition<br>StepOne Real Time PCR System for qPCR<br>xCELLigence RTCA MP Real Time Cell Analyzer for cell proliferation<br>FACS DiVA for flow cytometry<br>Prism 7 and 8 Graphpad Software
Data analysis	Zen Blue and Zen Black for image processing<br>ImageJ for NET quantification<br>FlowJo 9 an 10 for analysis of flow cytometry data<br>Scaffold 4 for proteomic data processing<br>R for generating a heat map<br>StepOne Real Time PCR System for running qPCR<br>xCELLigence RTCA MP Real Time Cell Analyzer for measuring cell proliferation..<br>Statistical analysis using Prism 7 and 8 Graphpad Software

For manuscripts utilizing custom algorithms or software that are central to the research but not yet described in published literature, software must be made available to editors and reviewers. We strongly encourage code deposition in a community repository (e.g. GitHub). See the Nature Research guidelines for submitting code & software for further information.

Data

Policy information about availability of data

All manuscripts must include a data availability statement. This statement should provide the following information, where applicable:

- Accession codes, unique identifiers, or web links for publicly available datasets
- A list of figures that have associated raw data
- A description of any restrictions on data availability

Data availability statement: Publicly available data were obtained from TCGA and the GTEx projects and the GEO repository (accession number GSE42605). The data that support the findings of this study are available within the article, supplementary information and supplementary data files and can be requested from the authors upon reasonable request.

Field-specific reporting

Please select the one below that is the best fit for your research. If you are not sure, read the appropriate sections before making your selection.

[x] Life sciences [ ] Behavioural & social sciences [ ] Ecological, evolutionary & environmental sciences

For a reference copy of the document with all sections, see nature.com/documents/nr-reporting-summary-flat.pdf

Life sciences study design

All studies must disclose on these points even when the disclosure is negative.

Sample size	For in vitro experiments no statistical methods were used to determine sample size, however, we performed at least 3 biological replicates with multiple technical replicates for every treatment condition (as stated in figure legends). In some experiments, sample number was increased to improve statistical power.<br><br>Generally, in vivo tumour models included 7-8 mice per treatment group. For all other in vivo studies, we performed assays on at least 4 mice in each treatment group. The number of mice used in each experiment is stated in the respective figure legends. Sample sizes were calculated based on previous experience and a priori power analysis (G* Power).
Data exclusions	No data were excluded
Replication	All the experiments were replicated. The number of replicates is stated in figure legends for each figure. This includes the number of biological replicates (n number) and technical replicates performed.
Randomization	For all ex vivo experiments, neutrophils were isolated from mice of different sexes and ages.<br>For spontaneous tumor models (skin and pancreatic), mice of various ages and sexes were used and randomly allocated to treatment groups.<br>Randomization was not possible for in vitro assays involving fibroblast and tumor cell lines.
Blinding	Where possible, technicians performing the experiment were blinded to experimental groups and treatments.<br><br>For all assays, the investigators were not blinded to group allocation during experiments as data collection, analysis and outcome assessment required that the investigators were not blinded.

Reporting for specific materials, systems and methods

We require information from authors about some types of materials, experimental systems and methods used in many studies. Here, indicate whether each material, system or method listed is relevant to your study. If you are not sure if a list item applies to your research, read the appropriate section before selecting a response.

Materials & experimental systems

n/a	Involved in the study	
[ ]	[x]	Antibodies
[ ]	[x]	Eukaryotic cell lines
[x]	[ ]	Palaeontology and archaeology
[ ]	[x]	Animals and other organisms
[ ]	[x]	Human research participants
[x]	[ ]	Clinical data
[x]	[ ]	Dual use research of concern

Methods

n/a	Involved in the study	
[x]	[ ]	ChIP-seq
[ ]	[x]	Flow cytometry
[x]	[ ]	MRI-based neuroimaging

Antibodies

Antibodies used	All antibodies are listed in Materials and Methods<br><br>Flow cytometry antibodies:<br><br>Ly6G FITC (Rat, Biolegend, clone: 1A8, dilution: 1:300, catalogue no: 127605)<br>CD45 PE (Rat, Biolegend, clone: 30-F11, dilution: 1:300, catalogue no: 103106)<br>CD45 FITC (Rat, Biolegend, clone: 30-F11, dilution: 1:300 catalogue no: 103108)<br>CD11b 647 (Rat, Biolegend, clone: M1/70, dilution: 1:300, catalogue no: 101218)<br>CD11b FITC (Rat, Biolegend, clone: M1/70, dilution: 1:300, catalogue no:101205)<br>CD11c PE-Cy7 (Armenian Hamster, Biolegend, clone: N418 , dilution: 1:300, catalogue no: 117318)<br>CD18 PE (Rat, Biolegend, clone: M18/2 , dilution: 1:300, catalogue no:101407)<br>CD62L APC (Rat, Biolegend, clone: MEL-14 , dilution: 1:300, catalogue no: 104411)<br>CD35 (Rat, Biolegend, clone: 7E9 , dilution: 1:300, catalogue no: 123407)<br>CD63 APC (Rat, Biolegend, clone: NVG-2 , dilution: 1:300, catalogue no: 143905)<br>F4/80 APC-Cy7 (Rat, Biolegend, clone: BM8 , dilution: 1:300, catalogue no: 123117)<br>PDGFRa PE (Rat, Biolegend, clone: APA5, dilution: 1:300 , catalogue no: 135905)<br>Thy1 APC-Cy7 (Rat, Biolegend, clone: 30-H12, dilution: 1:300, catalogue no: 105328)<br>Thy1 APC (Rat, Biolegend, clone: 30-H12, dilution: 1:300, catalogue no: 105311)<br>CD31 PE-Cy7 (Rat, eBioscience, clone: 390, dilution 1:300, 25-0311-82)<br>PDPN APC (Syrian Hamster, Biolegend, clone: 8.1.1, dilution 1:300, catalogue no: 127410)<br>EpCAM PE (Rat, eBioscience, clone: G8.8, dilution 1:300, catalogue no: 118205)<br><br>IF antibodies:<br><br>PDPN (Syrian Hamster, Biolegend, clone: 8.1.1, dilution: 1:100, catalogue no: 127402)<br>MPO (Goat, R&D Systems, clone: AF3667, dilution: 1:1000, catalogue no: AF3667)<br>Citrulline histone H3 (Rabbit, Abcam, clone: ab5103, dilution: 1:500, catalogue no: ab5103)<br>Anti-Alzheimer Precursor Protein A4 (Mouse, Merck, clone: 22C11, dilution: 1:100, catalogue no: MAB348A4)<br>Collagen I (Rabbit, Bio-Rad, clone: 2150-1410, dilution: 1:100, catalogue no: 2150-1410)<br>aSMA (Mouse, RnD Systems, clone 1A4, dilution: 1:100, catalogue no: MAB1420)
Validation	All antibodies obtained from commercial sources, with extensive validation for each application by the vendor, and referenced in literature. Antibodies have also been tested used extensively in other studies within the lab as well as several pilot studies.

Eukaryotic cell lines

Policy information about cell lines

Cell line source(s)	Skin CAFs and tumour cells isolated from Tyr::CreER; BrafCA; Ptenlox/lox primary skin tumours.<br>Lung CAFs isolated from inducible LSLKrasG12D/+;p53LSL-R270H/ER primary lung tumours.<br>Pancreatic CAFs and tumour cells isolated from LSL-KrasG12D/+;LSLTp53R172H/+;Pdx-1-Cre primary pancreatic tumours.<br>Matched healthy fibroblasts were taken from healthy skin, lung and pancreas of mice on the same genetic background as the tumour models.<br>Neutrophils were isolated from bone marrow of C57BL/6 mice.<br>C57BL/6 derived B16 F10 melanoma cell line was purchased from American Type Culture Collection (ATCC)
Authentication	Not performed
Mycoplasma contamination	No mycoplasma detected in established cell lines.
Commonly misidentified lines<br>(See ICLAC register)	No commonly misidentified cell lines were used.

Animals and other organisms

Policy information about studies involving animals; ARRIVE guidelines recommended for reporting animal research

Laboratory animals	C57BL/6 WT (bred in house) - For ex vivo experiments, 2-8 month old male and female mice were used. For in vivo experiments, 8-12 week old female mice were used.<br><br>LSL-KrasG12D/+;LSLTp53R172H/+;Pdx-1-Cre (provided by Tobias Janowitz; Hingorani SR et al. Cancer Cell. 2005;7(5):469-83 and Kraman M, et al. Science. 2010;330(6005):827-30). 8-32 week old male and female mice were used for these experiments.<br><br>LSLKrasG12D/+;p53LSL-R270H/ER (provided by Carla Martins; Kerr EM et al. Nature. 2016;531(7592):110-113).<br><br>Tyr::CreER; BrafCA; Ptenlox/lox (Purchased from The Jackson Laboratory; stock number 013590). 8-32 week old male and female mice were used for these experiments.

	PAD4KO and littermate controls (provided by Markus Hoffmann). 16-20 week old female mice were used in these experiments.
Wild animals	No wild animals were used in this study.
Field-collected samples	This study did not involve sample collection from fields.
Ethics oversight	All experiments involving animals were performed in accordance with UK Home Office regulations (PPL 80/2574 or PPL P88378375), and in the process were subject review and approval by the Medical research Council Laboratory of Molecular Biology (PPL 80/2574 or PPL P8837835) Animal Welfare and Ethical Review Bodies (AWERB).

Note that full information on the approval of the study protocol must also be provided in the manuscript.

Human research participants

Policy information about studies involving human research participants

Population characteristics	N/A
Recruitment	N/A
Ethics oversight	Research was conducted in full accordance with the Guideline for Good Clinical Practice and the Declaration of Helsinki. All biological materials used in this study and their subsequent evaluations were in accordance with the informed consent agreements obtained from all subjects.<br><br>Human tissue and plasma samples from patients with advanced melanoma (AJCC clinical stage III and IV) were kindly provided by the MELRESIST study investigators (UK NRES committee North East – Newcastle & North Tyneside 2 Research Ethics committee review reference 11/NE/0312). Control plasma was isolated from consenting healthy volunteers from the Cambridge Blood Centre. Human tissue microarrays for pancreatic adenocarcinoma were obtained from a commercial supplier (Biomax.US) and additional local ethical approval was obtained for use of these TMAs in our study (HBREC 2019.16).<br><br>This is included in the Methods section

Note that full information on the approval of the study protocol must also be provided in the manuscript.

Flow Cytometry

Plots

Confirm that:

- [ ] The axis labels state the marker and fluorochrome used (e.g. CD4-FITC).
- [x] The axis scales are clearly visible. Include numbers along axes only for bottom left plot of group (a 'group' is an analysis of identical markers).
- [x] All plots are contour plots with outliers or pseudocolor plots.
- [x] A numerical value for number of cells or percentage (with statistics) is provided.

Methodology

Sample preparation	As described in the Methods:<br><br>Tumors were minced using a razor and digested with 1mg/ml collagenase A and collagenase D and 0.4mg/ml DNase I in PBS at 37°C for 2h with rotation at 600rpm. 10mM EDTA was then added to stop the enzymatic reaction. The cell suspension was passed through a 70µm filter and stained with live/dead fixable violet stain (Thermofisher Scientific). Cells were subsequently stained with the following fluorescently conjugated antibodies; CD45 (30-F11), Ly6G (1A8), F4/80 (BM8), CD11b (M/170), CD11c (N418), Thy1 (30-H12), Podoplanin (8.1.1.), PDGFRα (APA5; all from Biolegend) and CD31 (390; eBioscience) at 1:300 dilution. Unstained and single-stained compensation beads (Invitrogen) were run alongside to serve as controls.<br><br>For bone marrow neutrophils, cells were aspirated from the femurs and tibias of C57BL/6 WT mice or tumour bearing mice and separated from the rest of the bone marrow using a Histopaque 1077/1119 gradient. The cells were harvested from the interface of the two components of the gradient and stained as above with antibodies.
Instrument	Flow cytometry was performed on LSR Fortessa (BD Biosciences) analyzers.
Software	Offline analysis was carried out on FlowJo (Treestar).
Cell population abundance	N/A - sorting was not performed
Gating strategy	All cell populations were characterised by first excluding debris (using SSC-A and FSC-A) and then gating on single cells (using FSC-A and FSC-H to distinguish singlets and doublets). Dead cells were excluded by gating on LIVE/DEAD® Fixable Violet Cell Stain. Cell populations were distinguished using the following antibody combinations:<br>Immune cells: CD45+

Myeloid cells: CD45+CD11b+
Neutrophils: CD45+CD11b+Ly6G+
Macrophages: CD45+CD11b+F4/80+
CAFs: CD45-CD31-EpCAM-Thy1+ or PDPN+ or PDGFRa+
Activated neutrophils: CD45+CD11b+Ly6G+CD18+CD62L+ (gMFI of CD11b, CD18 and CD62L)
Degranulating neutrophils: CD45+CD11b+Ly6G+CD35+CD63+ (gMFI of CD35 and CD63)
Apoptotic neutrophils: CD45+CD11b+Ly6G+7-AAD+AnnexinV+
Neutrophil ROS: CD45+CD11b+Ly6G+DCFDA+ (gMFI DCFDA)
Neutrophil Phagocytosis: CD45+CD11b+Ly6G+Green beads+ (gMFI beads)

☒ Tick this box to confirm that a figure exemplifying the gating strategy is provided in the Supplementary Information.
