# Supplementary material for: Stromal-driven and Amyloid β-dependent induction of neutrophil extracellular traps modulates tumor growth
Source: Nat Commun. 2021 Jan 29;12:683. doi: 10.1038/s41467-021-20982-2 (PMC7846803; doi:10.1038/s41467-021-20982-2)
Supplement: Supplementary file 4 — Description of Additional Supplementary Files [file 41467_2021_20982_MOESM4_ESM.pdf]

Description of Additional Supplementary Files

Supplementary Movie 1.

Neutrophil stained with SYTOX green undergoing NETosis. NET release is marked by a “sudden haze” of green material.
